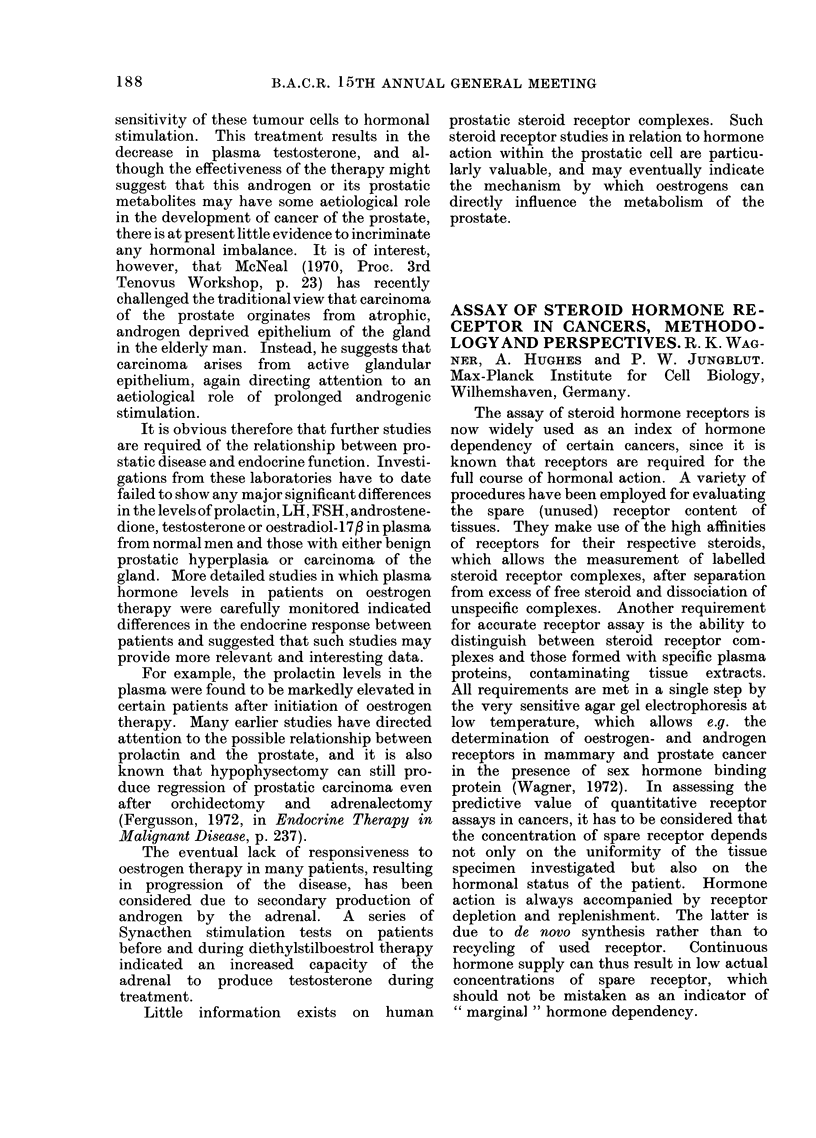# Proceedings: Assay of steroid hormone receptor in cancers, methodology and perspectives.

**DOI:** 10.1038/bjc.1974.177

**Published:** 1974-08

**Authors:** R. K. Wagner, A. Hughes, P. W. Jungblut


### ASSAY OF STEROID HORMONE RECEPTOR IN CANCERS, METHODOLOGY AND PERSPECTIVES.

R. K. WAGNER, A. HUGHES and P. W. JUNGBLUT. Max-Planck Institute for Cell Biology, Wilhemshaven, Germany.

The assay of steroid hormone receptors is now widely used as an index of hormone dependency of certain cancers, since it is known that receptors are required for the full course of hormonal action. A variety of procedures have been employed for evaluating the spare (unused) receptor content of tissues. They make use of the high affinities of receptors for their respective steroids, which allows the measurement of labelled steroid receptor complexes, after separation from excess of free steroid and dissociation of unspecific complexes. Another requirement for accurate receptor assay is the ability to distinguish between steroid receptor complexes and those formed with specific plasma proteins, contaminating tissue extracts. All requirements are met in a single step by the very sensitive agar gel electrophoresis at low temperature, which allows *e.g.* the determination of oestrogen- and androgen receptors in mammary and prostate cancer in the presence of sex hormone binding protein (Wagner, 1972). In assessing the predictive value of quantitative receptor assays in cancers, it has to be considered that the concentration of spare receptor depends not only on the uniformity of the tissue specimen investigated but also on the hormonal status of the patient. Hormone action is always accompanied by receptor depletion and replenishment. The latter is due to *de novo* synthesis rather than to recycling of used receptor. Continuous hormone supply can thus result in low actual concentrations of spare receptor, which should not be mistaken as an indicator of " marginal " hormone dependency.